# CAR‐T Cells: Current Status, Challenges, and Future Prospects

**DOI:** 10.1002/mco2.70606

**Published:** 2026-04-17

**Authors:** Aya Sedky Adly, Guillaume Cartron, Afnan Sedky Adly, Jean‐Christophe Egea, Pierre‐Yves Collart Dutilleul, Mahmoud Sedky Adly, Martin Villalba

**Affiliations:** ^1^ IRMB Univ Montpellier, INSERM Montpellier France; ^2^ Hospital Center Univ Montpellier Montpellier France; ^3^ LBN Univ Montpellier Montpellier France; ^4^ CSERD CHU Montpellier Montpellier France; ^5^ UFR Odontologie Univ Montpellier Montpellier France; ^6^ Royal College of Surgeons of Edinburgh Scotland UK

**Keywords:** chimeric antigen receptor, trogocytosis, mechanisms, challenges, algorithm, and machine learning

## Abstract

As chimeric antigen receptor (CAR)‐T cell therapy has expanded rapidly to meet the growing global cancer burden; many challenges have emerged as a critical factor influencing its efficacy. However, due to the complicated mechanisms of CAR‐T cells, human interference alone was insufficient to optimize the outcomes. In parallel, artificial intelligence (AI) has begun to intersect with CAR‐T cells, offering novel computational interferences that can refine therapeutic mechanisms. The literature is still lacking a comprehensive investigation that merges CAR‐T cell mechanistic biology and limitations with the advancing abilities of AI to meet these barriers. This review provides an overview of the mechanistic foundations of CAR‐T cell. It also investigates the various challenges facing the current CAR‐T therapies including toxicity, resistance, and accessibility issues. On this basis, we examined the way AI‐based innovations are being utilized to optimize the CAR‐T engineering and clinical management. Finally, we examined clinical studies and case studies incorporating AI elements, emphasizing both therapeutic mechanisms and outcomes of the study. By integrating mechanistic biology with computational innovation, this review provides a unified unique perspective that can guide the development of safer and more effective CAR‐T therapies.

## Graphical abstract

This graphical abstract outlines the current status, challenges, and future prospects of CAR‐T cells. The biological basis of CAR‐T cell therapy is the elegant redirection of adaptive immunity. Its initial successes have exposed a landscape of multifaceted challenges. Artificial intelligence turned its current challenges into tractable engineering problems. Bridging between basic science and clinical practice becomes a necessity to face the current limitations.

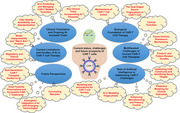

## 1 Introduction

In 1891, an American surgeon Dr William B. Coley made the striking discovery that tumors can shrink when the immune system is activated. However, the subsequent period of radiotherapy and chemotherapy hindered efforts to fully utilize the potential of immunotherapy. Only in the last two decades, the field of immunotherapy has experienced significant growth as a result of the growing comprehension of the immune systems and the cellular and molecular mechanisms behind the development of tumors. “Pushing the pedal” and “taking the breaks off” of the immune system are two novel therapies that have demonstrated tremendous potential in this field. Adoptive cell therapy and immuno‐checkpoint inhibitors are two treatments that have shown promise in treating a range of cancers; the former has primarily been used for solid tumors, while the latter has been used for hematological cancers [1]. Ex vivo engineering of the person's own immune cells to boost antitumors immunity is known as adoptive cell therapy. It consists primarily of three steps. To boost their anticancer activity, autologous immune cells are first extracted from the patient's peripheral tumor or blood tissues and then expanded and/or manipulated ex vivo. Last, the patient is reinfused with the altered cells to promote tumor regressions. Adoptive cell treatment has advantages over other cancer immunotherapies, which depends on the host's intrinsic antitumor lymphocytes, such as sufficient amounts of effector cells and low activity, which gives short‐lasting responses. More significantly, adoptive cell therapy can avoid the issues raised by individual variations in conventional treatment options because it is a personalized medicine. As a result, numerous engineering techniques have been looked into to raise adoptive cell therapy's safety and specificity levels [2]. The groundwork for chimeric antigen receptor (CAR)‐T treatment was established in the 1980s. Early investigations focused on altering T cells to express tumor‐specific receptors [3]. The initial idea of a synthetic receptor that combines the antigen‐binding variable regions (VH/VL) of a monoclonal antibody (mAb) with the signaling constant regions of the T cell receptor (TCR) was first reported by Japanese immunologist Dr Yoshihisa Kuwana and his team in 1987 [4]. In 1989, CAR‐T cell technology has been initially introduced via fusion of different areas of mAbs light and heavy chains to the TCR constant regions. The synthetic receptors have the ability to identify tumor antigens [5] and T cells are trained to target the tumor cells, which express such antigens. Intracellular signaling is triggered causing T cells activation [6]. Unlike previous adoptive cell therapies, CAR‐T cells are armed with synthetic CAR receptors [7] and five generations of CAR molecules have passed since then. The first‐generation CARs were developed in 1990 and featured only the CD3z chain as their intracellular signaling domain, which provided initial activation but resulted in limited persistence and efficacy [8]. To address this, in the 2000s, second‐generation CARs incorporated a single costimulatory domain such as CD28 alongside CD3z, which significantly enhanced T cells proliferation, cytokine production, and in vivo persistence [9]. Third‐generation CARs combined two costimulatory domains with CD3z in a single receptor synergizing signaling for further improvement [10].

The fourth generation, or the T cells redirected for universal cytokine‐mediated killing, emerged around 2010 and are based on a second‐generation CAR but are engineered to release transgenic immune modulators, such as IL‐12 cytokines, upon antigen recognition to alter the tumor microenvironment [11]. Finally, fifth‐generation CARs were built upon the second‐generation design by incorporating truncated cytokine receptors (e.g., IL‐2 receptor β‐chain fragment) that activate the Janus kinase/signal transducer and activator of transcription (JAK/STAT) pathway upon CAR engagement, creating a fully integrated signaling cascade that aims to further augment T cell expansion and prevent exhaustion [12].

CAR‐T cell therapy has started a new era of precision immunotherapy, leveraging well‐characterized mechanisms such as perforin–granzyme cytotoxicity, Fas–FasL signaling, and cytokine‐driven immune recruitment [13]. More recently, trogocytosis has emerged as a significant modulatory process, which can determine the effects of therapy and resistance through enabling antigen transfer mechanisms between tumor and immune cells [14]. Understanding these dynamic mechanisms continue to be essential in the design of CAR‐T cells and optimization of clinical efficacy [15]. CAR‐T cell therapy has demonstrated significant clinical achievements, especially in the management of hematological malignancies, leading to the United States Food and Drug Administration approval of several products targeting antigens such as CD19. Such therapies have induced deep and durable remissions in a significant proportion of patients who had exhausted all conventional treatment options [16, 17].

Currently, artificial intelligence (AI) has begun to intersect with this revolution by proposing computational interferences to guide CAR construction, improve antigen recognition, and predict cellular responses in silico [18]. AI has brought forth a multicore era of cancer treatment, owing to the remarkable advancements in bioinformatics and tumor immunology. The quality and efficiency of CAR‐T cells therapies have increased owing to the ongoing development of AI and machine learning techniques and the growing of computational power [19, 20]. Trogocytosis role in CAR‐T cell therapies are also progressively receiving more attention. Efforts were made for improving AI outcomes in CAR‐T cells through overcoming trogocytosis, that possesses a challenging task due to the complicated nature of some types of tumors [21].

Despite the recent innovative developments, there are still significant unmet medical needs. The limited effectiveness of CAR‐T cells in solid tumors [22] and the life‐threatening toxicities remain a significant concern [23]. The trogocytosis phenomena, which comprises a bidirectional membrane fragments exchange between the cells, can negatively influence the CAR‐T cell therapies efficacy [24]. Other limitations include the complexity of manufacturing personalized and accessible CAR‐T treatments, as well as the limited persistence of CAR‐T cells in some patients causing disease recurrence [25]. This review aims to provide a comprehensive perspective on CAR‐T cell mechanistic biology and all the challenges impeding its full therapeutic potential, while evaluating how emerging AI approaches can bring transformative solutions across design, manufacturing, and clinical application. By integrating mechanistic insights with biological, clinical, and logistical barriers, we highlight the unresolved gaps that continue to restrict safety, accessibility, and efficacy. Furthermore, this work examined the growing evidence supporting AI‐driven innovations that enhance prediction, optimization, and translation of CAR‐T therapies.

The manuscript can be divided into seven parts in addition to the introduction. First, we discussed the biological foundation of CAR‐T cell therapy. Second, the multifaceted challenges of current CAR‐T cell therapies were explored. Third, the integrating role of AI in addressing CAR‐T challenges were discussed. Fourth, clinical translation and ongoing AI‐assisted trials were investigated. Fifth, the current limitations and hurdles of AI in CAR‐T cell therapy have been provided. Sixth, future perspectives were presented. Finally, the concluding remarks were summarized.

## 2 The Biological Foundation of CAR‐T Cell Therapy

The biological basis of CAR‐T cell therapy is the elegant redirection of adaptive immunity, but recent research has increased our understanding of the critical cellular and molecular determinants of efficacy and failure. While the core principle remains to engineer autologous T cells with a synthetic CAR for major histocompatibility complex (MHC)‐independent antigen recognition, current research focuses on the functional quality of the starting T cell population, the complex signaling kinetics of advanced CAR constructs, and the postinfusion biology of CAR‐T cell persistence and differentiation [26, 27].

### 2.1 Core Mechanisms of CAR‐T Cell Action

#### 2.1.1 Antigen Recognition and Activation

CAR‐T cells cytotoxic mechanism is considered similar to the natural T cells. When a patient receives CAR‐T cells infusion, the CAR receptors single‐chain variable fragments (scFv) exposes the patient's T cells to antigens expressed by the tumor [28]. Conformational changes and activation occur in CAR‐T cells directly after their interaction with the antigen. The intracellular domain's constituent parts organize into microclusters through centripetal motion to create the immunological synapse core regions, which facilitates the phosphorylation and recruitment of the downstream cascade proteins. After activation, the CAR‐T cells undergo through an extended differentiation and proliferation processes, that is crucial for an effector function or cancer‐killing activity of a CAR‐T cell [29].

#### 2.1.2 Cytotoxic Mechanisms

CAR‐T cells can achieve cancer cells killing by three main synergistic mechanisms, which involve the perforin–granzyme systems, the Fas–Fas ligand (FasL) axis, and other immune systems recruitment through cytokine secretion [6].

#### 2.1.3 Perforin–Granzyme Pathway

**FIGURE 1:**
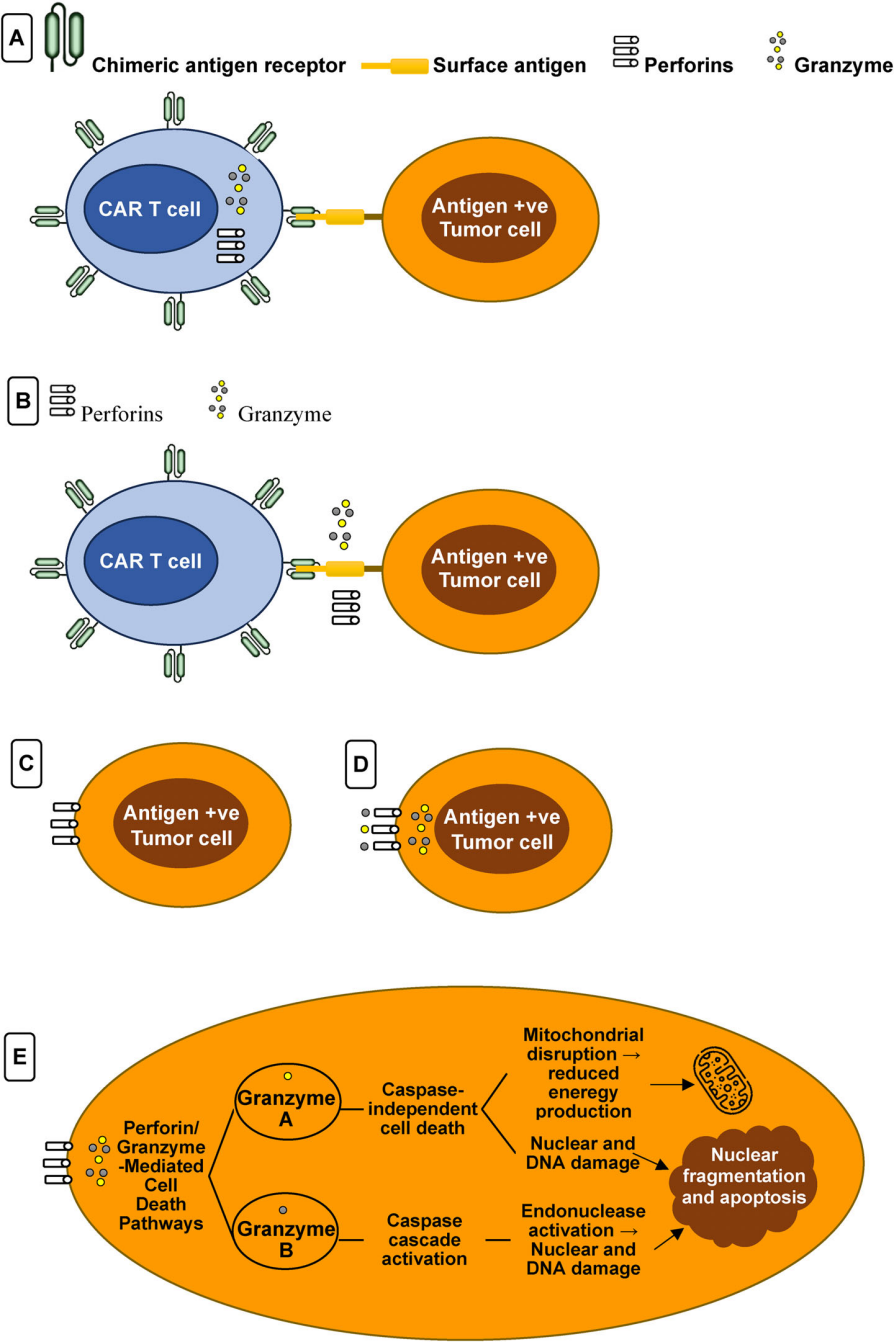
Perforin and granzyme pathway. (A) Surface antigens on target tumor cells are recognized by the specific chimeric antigen receptor on CAR‐T cells. (B) Degranulation of lytic granules inside CAR‐T cells, which contain perforins and granzyme. (C) Perforins attach themselves to the plasma membrane of the tumor cell. (D) Perforins allow entrance of granzyme into the cytosol of the target cell. (E) Entry of granzymes induces apoptotic cell death by caspase‐independent cell death (granzyme A) or caspase cascade activation (granzyme A).

The first mechanism for CAR‐T cell‐induced cancer cells lysis is the perforin and granzyme pathway as shown in Figure 1. When CAR‐T cells are activated and surface antigens over a target T cell are recognized, the CAR‐T cell lytic granules quickly degranulate, releasing the cytotoxic effector proteins (granzymes and perforin) [30]. Following their release, perforin causes transmembrane holes to form on the tumor cells’ plasma membranes, giving cytotoxic granzymes entry into the cytoplasm. Granzyme can mediate apoptotic cell death after it enters the tumor cell's cytoplasm by cleaving the necessary substrates [31] and can trigger caspase‐independent and caspase‐dependent apoptotic pathways, which destruct antigen‐positive tumor cells. In the end, the neighboring phagocytic cells will quickly eliminate the dead cancer cells [32].

#### 2.1.4 FasL and Tumor Necrosis Factor‐Related Apoptosis‐Inducing Ligand Pathways

**FIGURE 2:**
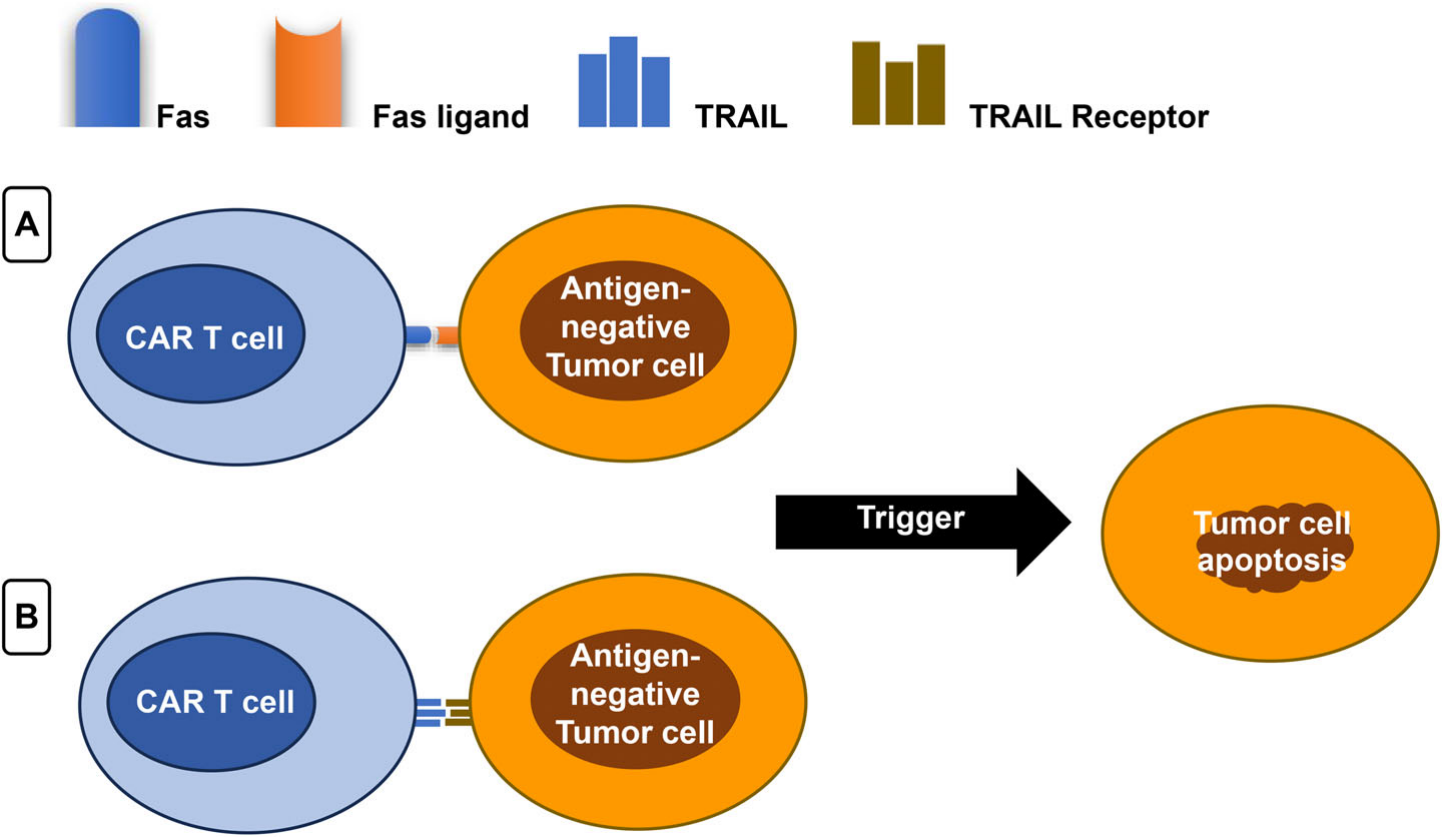
An illustration of the CAR‐T cell cytotoxicity through death ligand–death receptors that is not dependent on perforin and include two similar mechanisms: (A) Fas–Fas ligand axis utilizing Fas on CAR‐T cell that binds to Fas ligand on the tumor cell. (B) TRAIL system utilizing TRAIL on CAR‐T cell that binds to Fas ligand on the TRAIL receptor.

CAR‐T cells can also utilize death ligand–death receptors including the FasL axis and tumor necrosis factor‐related apoptosis‐inducing ligand (TRAIL) systems as shown in Figure 2. Fas–FasL‐induced cytotoxicity, which is not dependent on perforin, occurs when Fas in a target T cell membrane would bind to FasL on CAR‐T cell, which is activated [33]. Caspase 8 is triggered when FasL trimerizes the Fas receptor. This allows procaspase 3 to mature into mature caspase 3, that would then mediate tumors cells destruction via downstream pathway. In contrary to the perforin–granzyme axis, a Fas–FasL systems have been shown to be a slower mechanism, which is needed for targeting the cells of the antigen‐negative cancer within antigen‐positive cancer microenvironments. Furthermore, the interactions among CAR‐T cells and tumors cells via Fas/FasL might either increase the antitumor capacity or decrease tumor escape resulted from heterogeneous antigen expressions [34].

#### 2.1.5 Indirect Cytotoxic Mechanisms

**FIGURE 3:**
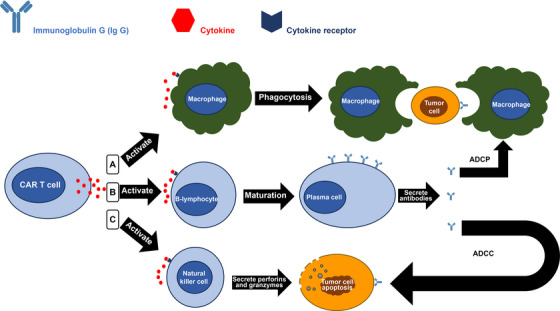
Cytokine secretion by CAR‐T cell recruiting more immune system elements including: (A) macrophages activation for phagocytosis, (B) B‐lymphocyte maturation into plasma cell and secreting antibodies, which induce both antibody‐dependent cellular phagocytosis (ADCP) and antibody‐dependent cellular cytotoxicity (ADCC) on tumor cells. (C) Natural killer cells that secrete perforins (which perforate the membrane of the tumor cell) and granzymes (which enters the cytosol and induce apoptosis).

The other CAR‐T cells mechanisms that could kill cancer cells are by recruiting extra immune system elements to enter tumor cells and kill cancer cells. This was done via the activated CAR‐T cells. Those activated cells produce more cytokines, that boost antitumoral activities as shown in Figure 3. The cells’ release of cytokines is essential for causing tumor lysis because it triggers stromal sensitization, polarization of macrophages, and CAR‐T cell death. Interleukin‐12 (IL‐12) in particular has been demonstrated to stimulate antitumor immune responses [35]. Additionally, the process is linked to increased T cell cytolytic activities, innate immune cell activation and recruitment, and immune suppressor cell reprogramming associated with the stroma. Additionally, dead cancer cells had the ability to release cytokines, which promoted the growth of CAR‐T cell, which in turn destroy tumors cells. Additionally, CAR‐T cells enhance their antitumors potential by attracting additional immune cells (like B‐cells and Natural Killer (NK)  cells), to the cancer sites through the use of cytokines [28]. Finally, CAR‐T cells possessed the capacity to completely destroy tumors cells, either momentarily or permanently—a state referred to as remission. Certain types of blood cancer may experience a long‐term remission as a consequence of CAR‐T cells treatments, which could stay in a body for long periods after their infusion [36].

### 2.2 The Dual‐Edged Sword: Trogocytosis in CAR‐T Cell Therapy

- The process of trogocytosing membrane segments that bear antigen/MHC from antigen‐presenting cells (APCs) is an active process for T cells. This process necessitates actin cytoskeleton reorganization and TCR signaling. At the immunological synapse, ligands for costimulatory receptors and antigen/MHC complexes are acquired. It has been demonstrated that the T cell plasma membrane can be pouched and a fragment of the APC membrane and cytoplasm is gnawed at the central supramolecular activation cluster, which is the location of higher accumulation of antigen/MHC and bound TCRs on the opposing APC side of the immunological synapse [37].
- TCR‐mediated trogocytosis necessitates actin cytoskeleton reorganization and TCR activation to cause the APC membranes and T cells to zipper. Because of this, trogocytosis was described as frustrated phagocytosis; in fact, cells with the ability to execute trogocytosis, including T cells, are also capable of phagocytosis [38].
- A growing amount of data indicates that T cell trogocytosis is not only a common occurrence in vivo but also plays a significant role in immunological regulation and intercellular communication. Studies conducted in vitro have revealed a mechanism that is qualitatively comparable to phagocytosis [39].
- Through membrane‐associated molecules acquisition, trogocytosis has been demonstrated to transfer new functional abilities from one cell type to another [40].
- Through the process of trogocytosis, cytotoxic T lymphocytes (CTLs) can transfer their TCRs to recipient CTLs with varying clonotypic specificity. However, it may be related to the duration and strength of TCR stimulation, the exact mechanism causing T effector cell polarization is yet unknown. Obtaining donor TCRs allows for the recognition of extra antigen and the growth of virus‐specific clones without the need for proliferation [41].
- CAR‐T cells trogocytic acquisition of target antigens can decrease target density on tumor cells as well as promoting “fratricidal” (mutual) CAR‐T cells death and exhaustion [42].
- CD8+ T cells trogocytosis can take place by either attacking target cells including the tumor cells or once APCs prime CD8+ T cells. During their contact with APCs, CD8+ T cells strip peptide MHC class I (pMHC‐I) complexes from these APCs. This may promote the growth of high‐affinity TCR‐producing CD8+ T cells in a selected manner, ultimately leading to the maturation of these cells. CD8+ T cells as well strip pMHC‐I complexes from the tumor cells. Consequently, CD8+ T cells trogocytosis can have a suppressive action in tumors immune responses [43, 44].

## 3 The Multifaceted Challenges of Current CAR‐T Cell Therapies

The initial successes have exposed a landscape of multifaceted challenges that now define the current state of CAR‐T research and development [45]. These challenges are not isolated, but are intricately linked, ranging from severe, life‐threatening toxicities and burdensome manufacturing processes to fundamental biological barriers, which leads to antigen escape and relapse [46].

### 3.1 Clinical Toxicities

#### 3.1.1 Toxicities Induced by CAR‐T Therapies

Life‐threatening CAR‐T cell‐associated toxicities are considered from the main drawbacks of CAR‐T cell therapy that still need to be addressed despite its remarkable effectiveness [47]. The two most common toxicities linked to CAR‐T cells therapy are the cytokine‐release syndrome and the immune effector cell‐associated neurotoxicity syndrome. These toxicities might show up as supraphysiologic cytokine production causing fever and problems in the neurological, circulatory, respiratory, and digestive systems [48]. Systemic cytokine release toxic levels and severe immune cells cross‐activation in certain patients can cause elevated serum ferritin, massive in vivo T cell expansion, renal failure, hemophagocytosis, liver enzymes, pulmonary edema, splenomegaly, elevated cerebrospinal fluid cytokine levels, absence of NK cell activity, and disruption of the blood–brain barrier resulting in neurotoxicity from cerebral edema [49]. Low‐grade Cytokine Release Syndrome (CRS)  can be managed by antipyretics and intravenous fluid to counteract for the vascular leakage. However, caution should be made to avoid subsequent pulmonary edema [50]. Severe CRS can be managed by tocilizumab, which was found to be effective in controlling the symptoms within few hours without impairment of CAR‐T cell efficacy [51]. However, tocilizumab can raise the levels of interleukin‐6, which can lead to neurotoxicity [52] in addition to its ability to cause long‐term immunosuppression [53]. Corticosteroids with or without tocilizumab had been used for management of severe cases of CRS and Immune Effector Cell‐Associated Neurotoxicity Syndrome (ICANS)  [51]. However, the long‐term use of corticosteroids can affect the efficacy of CAR‐T cell while in short duration and low‐dose corticosteroids there was no adverse effect seen on these cells [54]. Anakinra (recombinant interleukin‐1 receptor antagonist) is currently used to treat refractory ICANS. Also, the prophylactic use of anakinra to prevent ICANS and CRS show promising results [51]. The immunosuppressive effect of anakinra can increase the risk of opportunistic infections; thus, antimicrobial prophylaxis should be put into consideration when using this drug [55]. Other possible therapies for refractory cases include: interleukin‐1R inhibitors, arctigenin, alemtuzumab, Tumor Necrosis Factor (TNF)‐α blockers, ibrutinib, Granulocyte‐Macrophage Colony Stimulating Factor (GM‐CSF) inhibition, and cyclophosphamide [48].

#### 3.1.2 CAR‐T Cells Mediated Cytotoxicity Against Normal Tissues, on Antigen Reduction (On‐Target, Off‐Tumor Effect)

The fact that the majority of possible target antigens are frequently coexpressed on nonmalignant tissues, poses significant risks of morbidity because of on‐target, off‐tumor toxicities, which significantly impede developments of CAR‐T cells of patients with solid tumors. Preclinical and clinical trials employing CAR‐T cells in antigens targeting, shared by malignant and nonmalignant tissues, have documented cases of on‐target, off‐tumor toxicities of variable severity. Developments of more specialized CAR systems, such as those that allow external control of T cell survival or function, has been the subject of interest in research. The probability of an on‐target, off‐tumor impact may be reduced by adjusting the CAR architecture and scFv affinity. Other methods involve engineering strategies intended for eliminations of CAR‐T cells in a timely manner or to exogenously modulate CAR‐T cells activity. It was formerly been explored to concentrate antitumor activity within tumor microenvironments through loco‐regional injection of CAR‐T cells, that is, loco‐regional injections of anti‐B7‐H3 CAR‐T cells have shown more‐effective antitumor response in teratoid tumors [56].

### 3.2 Tumor‐Intrinsic Resistance Mechanisms

#### 3.2.1 Antigen Reduction or Loss Following CAR‐T Cell Treatments

Antigen loss, which was seen in 30–70% of the patients, is the term used to describe the decrease or loss of target antigens expressions on tumor cells after CAR‐T cells therapies. CAR‐T cells could no longer successfully identify and target cancer cells when antigen loss takes place. Antigen‐losing patients are more likely to relapse. It also restricts these patients’ options for treatment. Antigen loss is a complex phenomenon that involves multiple contributing factors such as immune editing, clonal selections, antigens shedding, genetic mutations, and epigenetic modifications. The primary goals of counterstrategies for antigen loss are to enhance effectiveness of CAR‐T cells (via manufacturing CAR‐T cells, which could target multiple antigens concurrently), reduce the possibility of antigen loss (through targeting shared tumor‐associated antigens (TAAs), which have reduced liability to loss or reduction in CAR‐T cells treatment), and target alternative antigens, which are unlikely to be lost throughout therapy [57, 58, 59].

#### 3.2.2 Microenvironment Immunosuppression

Tumor microenvironment promotes angiogenesis in order to counteract the effects of hypoxic and acidic environments, supporting tumor survival, invasion, and metastasis, and encouraging the early proliferation of cancer cells at the onset of the tumor.

Due to the immunosuppressive cancer microenvironment, CAR‐T cells will be exhausted and will not activate sufficiently when they penetrate solid tumors [48]. For overcoming this challenge, a combination therapeutic strategy has been introduced, in which CAR‐T cells were administered alongside radiotherapy [60]. Additional methods include modifying CAR‐T cells to produce immune‐stimulatory cytokines or CARs resistant to immunosuppressive factors, removing or rerouting immune suppressor cells within the tumor microenvironment, interfering with inhibitory signaling pathways and immunosuppressive cytokines, and combining CAR T cells with immune checkpoint blockade [48].

#### 3.2.3 Tumor Heterogeneity

The difficulty in identification of the ideal target antigens is considered a key distinction between solid tumors and hematological cancers. Hematological cancers usually present heterogeneous targets; however, they also frequently express specific markers. TAAs, which are expressed at reduced levels in normal tissues and substantially expressed on the tumor itself, are also more frequently seen in solid tumors. Moreover, TAA heterogeneity between patients with the same disease and different tumor types (primary versus metastatic) is seen in solid tumors [61]. In order to address this issue, a number of techniques were developed, including the use of CARs that target adapter molecules to link different soluble antigen‐recognition moieties, the design of CAR‐T cells to target multiple TAAs, and the coexpression and secretion of bi‐specific T cell engagers (BiTEs) by CAR‐T cells [48].

### 3.3 Product‐ and Patient‐Related Hurdles

#### 3.3.1 Trafficking of CAR‐T Cells and Tumor Infiltrations

Early‐stage clinical research on CAR‐T cells treatments for solid cancers had demonstrated some anticancer effectiveness; nevertheless, specific obstacles, such as limited capability of the CAR‐T cell to migrate to and infiltrate solid cancers, still need to be solved [62]. Using delivery routes other than the systemic deliveries including local administration is a way to lessen those restrictions. Chemokine receptors expression on CAR‐T cell that bind to and react to cancer‐derived chemokines was a suggested strategy that can greatly improve CAR‐T cells trafficking. Engineering a CAR‐T cell to express heparinase for extracellular matrix degradation of tumor revealed antitumor activity and enhanced tumor infiltration [49].

#### 3.3.2 Immunogenicity of CAR‐T Cells

The host immune system recognition of CAR constructs can contribute to cytokine‐related toxicities and therefore, using humanized or human antibody fragments as an alternative to murine‐derived CARs to reduce CAR immunogenicity can be of great significance. Furthermore, it is possible to alter the hinge and/or transmembrane regions of CAR for reducing its immunogenicity; remarkably, this also enhances CAR‐T cell persistence [49].

#### 3.3.3 Patient Accessibility to CAR‐T Cells Therapy

Accessibility can be considered from the most difficult challenges, as only 1:5 of patients have accessibility to receive CAR‐T cell treatment [63]. Owing to the autologous nature of these therapies, there are drawbacks that impact accessibility. These include the procedure's high cost, manufacturing failures for certain patients, and the treatment's lengthy manufacturing process, which take more than 20 days to complete and cause a delay in treatment availability. These factors have resulted in a rise in interest in creation of so‐called “off‐the‐shelf” allogeneic cellular treatments, in which the T cells were derived of healthy donors cells instead of patients tissue [64].

### 3.4 Trogocytosis (The Hidden Driver of CAR‐T Cells Hyporesponsiveness and Dysfunction)

CAR‐mediated trogocytosis can be considered one of the most important challenges in CAR‐T cells therapy as it can weaken the CAR cells antitumoral functions via redirection of their effector functions against other CAR‐expressing cells, causing CAR cells exhaustion and fratricide [65]. Recently, trogocytosis has been shown to be a mechanism of CAR NK cells dysfunction in which CARs are targeting specific cancer antigens and constituting of many intracellular signaling domains [24]. Despite there is no particular management approach to regulate trogocytosis, strategies to overcome trogocytosis‐induced CAR cells exhaustion, fratricide, and antigen loss have the ability to increase CAR cells efficacy and tumors clearance. The potential approaches that have been introduced in the literature to modulate trogocytosis included pharmacological targeting, dual CAR strategy by harnessing an activating CAR with an inhibitory CAR, affinity modulation by lowering CAR antigen‐specific affinity without affecting efficacy, redesigning of armored CAR constructs for hampering trogocytosis, and modifying signaling domains through adjustment of the signaling domain impacted by trogocytosis [21]. In light of these multifactorial challenges, innovative solutions are required that extend beyond conventional biological engineering.

## 4 The Integrating Role of AI in Addressing CAR‐T Challenges

AI can serve as an integrating engine, synthesizing disparate data streams to build a predictive, personalized, and more effective future for CAR‐T cell therapy, turning its current challenges into tractable engineering problems [18]. It was revealed that AI re‐emerges as a central tool for addressing limitations at the interface of biology, engineering, and clinical implementation [66]. The proposed AI applications that have been reported in the literature for the formerly illustrated challenges are demonstrated in Table 1.

**TABLE 1:** Summary of the current challenges and limitations, management, and proposed AI applications of CAR‐T cell therapies in the literature.

Limitations	Management	Proposed AI applications
Toxicities induced by CAR‐T therapies	Antipyretics and intravenous fluid to counteract for the vascular leakage Tocilizumab Corticosteroids with or without tocilizumab Anakinra to treat refractory ICANS Interleukin‐1R inhibitors, arctigenin, alemtuzumab, TNF‐α blockers, ibrutinib, GM‐CSF inhibition, and cyclophosphamide for refractory cases [48]	AI models and simulations can identify patients with manageable toxicities or who might be at risk of relapse. This could allow for the planning of risk mitigation strategies, which could lower the mortality rate and complications associated with toxicity [67].
CAR‐T cells mediated cytotoxicities against normal tissues, on antigen reduction (on‐target, off‐tumor effect)	Adjusting the CAR architecture and single‐chain variable fragment affinity Engineering strategies intended to eliminate CAR‐T cells by timely manner or to exogenously modulate CAR‐T cells activity Concentrate antitumor activity within the tumors microenvironment through loco‐regional injection of CAR‐T cells [56]	Prediction models that enhance the accuracy of neoantigen prediction [68]
Antigen reduction or loss following CAR‐T cell treatment	Increase the effectiveness of CAR‐T cells Reduce the likelihood of antigen loss Target other antigens that are unlikely to be lost through treatments [57]	As accurate antigen expression analysis is crucial for assessing the effectiveness of CAR‐T cells and detection of antigen loss, AI was used to analyze the changes in antigen‐presenting pathways [69]. The mechanism of activating CAR‐T cells through antigen‐presenting beads and their consequent multiplication is established in a work that included reinforcement learning [70].
Microenvironment immunosuppression	CAR‐T cells were administered alongside radiotherapy. Modifying CAR‐T cells to produce either immune‐stimulatory cytokines or immunosuppressive factors‐resistant CARs Removing or rerouting immune suppressor cells within the tumor microenvironment Interfering with inhibitory signaling pathways and immunosuppressive cytokines Combining CAR T cells with immune checkpoint blockade [48]	Tumor microenvironment can be analyzed with AI [71]. Various models were created to examine different characteristics of CAR‐T cells therapies, such as machine learning models for gaining a thorough comprehension of the relative effects of tumor immunosuppressive environments, and proliferation of CAR‐T cells therapy [72, 73, 74].
Tumor heterogeneity	Use of CARs that target adapter molecules to link different soluble antigen‐recognition moieties The design of CAR‐T cells to target multiple TAAs The coexpressions and secretion of BiTEs by CAR‐T cells [48]	A study presented a model for detecting personalization of CAR‐T cells production using a machine learning classifier [75]. A clinical decision support system was proposed for improving the CAR‐T cell product's ability to be personalized for targeting [76].
CAR‐T cells trafficking and tumor infiltrations	Using delivery route other than systemic deliveries including local administrations Chemokine receptor expressions on the CAR‐T cell, which bind to and react to tumors‐derived chemokines [49]	Analyzing particular chemokine profiles by developing a machine learning‐based approach guided by meta‐analyses to help in patients receiving CAR‐T therapy [77]
Immunogenicity of CAR‐T cells	Using humanized or human antibody fragments as an alternative to murine‐derived CARs Alter the hinge and/or transmembrane regions of CAR for reducing its immunogenicity and enhance CAR‐T cell persistence [49]	Nine immunogenicity factors integrated by Neopepsee, which is a machine‐learning‐based neoantigen prediction algorithm [68]
Patient accessibility to CAR‐T cells therapy	Off‐the‐shelf allogeneic cellular treatments, where the T cells are derived from healthy donor cells instead of patient tissue [64]	One survey highlighted automated cell expansion trends and key performance indicators (KPIs) including artificial neural networks [78, 79]. The establishment of an integrated perfusion bioreactor can be considered a key element in increasing the manufacturing process as it is equipped with several sensors for deploying the AI‐supported control strategy. Moreover, it can enable cocultivation of CAR‐T cells as much as it is required. Integrating automation enabled solutions for routine laboratory operations is important for the accessibility process [80].
Trogocytosis‐induced CAR cells exhaustion and hyporesponsiveness	Pharmacological targeting Dual CAR strategy by harnessing an activating CAR with an inhibitory CAR Affinity modulation by lowering CAR antigen‐specific affinity without affecting efficacy Redesigning of armored CAR constructs for hampering trogocytosis Modifying signaling domains through adjustment of the signaling domain impacted by trogocytosis [21]	A highly dimensional reduction algorithms were utilized for detection of NK cell subsets, which degranulated after active trogocytosis performance [81, 82]. An algorithm for visualizations of t‐distributed stochastic neighbor embedding was used for further detection of tumor markers expressions that are presented on surfaces of NK cells as a result of trogocytosis [83]. A 3D fast tracking algorithm was developed for trogosome material tracking in microscopy [84].

### 4.1 In Silico Design and Discovery: Optimizing CAR Constructs and Identifying Novel Targets

The emergence of sophisticated in silico design and discovery is offering a powerful, rational framework to engineer superior CARs and discover novel therapeutic targets. By leveraging AI, structural bioinformatics, and machine learning on vast datasets of protein sequences, structures, and outcomes, researchers can now virtually optimize CAR constructs and identify new targets [85].

#### 4.1.1 Effectiveness, Enhancement, and Optimization of CAR Constructs

In order to enhance the effectiveness of CARs and increase their appropriateness for tumors, researchers are trying to enhance and optimize various CARs. In clinical practice, it might be difficult to rate the effectiveness of various “off‐the‐shelf” immunological treatments and to identify clinical responders. Meanwhile, the existing, labor‐intensive, expensive, and time‐consuming traditional methodologies used to evaluate efficacy, limit the ability of a researcher to find the best CAR architecture for the purpose of developing a prospective clinical application. The CTLs optimal function rely on the effectiveness of the immunological synapse [58]. Prior research indicates that CAR immunological synapse quality can serve as a functional indicator for prognosis in CAR‐T immunotherapies. However, clinically quantifying the quality of CAR‐T immunological synapse is difficult. Prior proposals have been made for CAR‐T immunological synapse quality quantification based on machine learning. A method was described for quantifying the CAR immunological synapse quality based on machine learning to determine the effectiveness of CAR‐modified cells. This method used machine learning‐based algorithms for quantifying the immunological synapse quality, imaging CAR immunological synapse, segmenting the CAR immunological synapse images and defining the CAR immunological synapse focal plane [86].

Another study proposed a mix of deep learning‐based segmentation and optical diffraction tomography to propose and experimentally evaluate an automated effectiveness assessment technique for immune synapse dynamics. A new avenue for immunological research is made possible by the suggested method, which permits an automated spatiotemporal study of immunological synapse kinetics related with immunological synapse dynamics [87].

A mathematical explanation model of T cell responses was created by Kirouac et al. in which tumor antigen engagement coordinately optimizes transitions between effector, memory, and exhausted T cell states. The main factors influencing clinical response are found to be cell‐intrinsic variations in memory cell turnover rate and effector cytotoxic efficacy. This model was trained utilizing clinical data that were gained from CAR‐T products in various hematological malignancies. Using a workflow for machine learning. This work showed that further pharmacological variances results from cellular level patient malignancies interactions, and product‐intrinsic variations can reliably anticipate possible patient outcomes according to the preinfusion transcriptomes [88].

A subset of machine learning called reinforcement learning may also be able to enhance and optimize other procedures such as producing therapeutic cells more effectively. The method of CAR‐T cells activation is formulated in another work that included reinforcement learning. Reinforcement learning agents are trained in the simulation to adjust the amount of the beads inside the culture in order to amplify the populations of effector cells inside the conclusion of the culture for the creation of therapeutic CAR‐T cell [70].

#### 4.1.2 Detection and Identification of Novel Targets

CARs have demonstrated encouraging therapeutic potential when used to target malignancies with low risk. However, even though the CAR‐T cell therapeutic method has presented several therapeutic advantages in many patients, there is a chance that the treatment will have serious side effects including CRS, which is linked directly to the activation of obviously potent immune system effector responses [89]. Analyzing particular chemokine and cytokine profiles that frequently show similarities between individuals can be used in detection of CRS. The aim of a prior study was to build a machine learning‐based approach guided by meta‐analyses to help in CRS detections of patients receiving CAR‐T treatment. In order to establish a meta‐review informed approach for the detection of CRS relying on certain cytokines peak concentration and data from former clinical research, approaches using machine learning algorithms are required [77].

Another study presented a model for detecting the required dose of T cell stimulation for personalization of CAR‐T cells production. A classification model was used to quantify the link between a blood sample of T cells and its CAR‐T cells products. Afterward, a model that generates the necessary simulation dose to attain the intended CAR‐T cell product phenotype was developed as a proof‐of‐concept. A random forest machine learning classifier detected whether the CAR‐T cells product was originated from healthy or patient T cells based only on phenotype, which is in accordance with the finding that CAR‐T cells acquired from healthy and patient samples were dissimilar phenotypically. As a result, distinct categorization models were created for samples obtained from patients and healthy individuals. In this study, the machine learning classifier was used to determine the relative relevance of each T cell feature in blood samples of either healthy or patient T cells [75].

A machine learning workflow was presented in another study that identifies spatial neighborhoods where T cells exhibit functional activation signatures and links those niches to durable responses. The same machine learning method and spatial proteomics pipeline can be applied to tissue sections containing CAR‐T cells (detectable by CAR or TCR tags) to find regions where CAR‐T cells show sustained activation markers or functional signatures, that is, candidate locations for prolonged activation [90].

A study by Song et al. observed significant methylation signatures to distinguish between CAR‐transduced and CAR‐untransduced T cells. In order to identify possible locations of the prolonged activation of the CAR‐T cells, this study combined machine learning computational techniques to mine CAR‐T cell‐specific methylation areas. Patients’ T cell methylation profiles associated with the B‐cell malignancies were thoroughly examined using a number of sophisticated machine learning algorithms. This work used a potent computational approach based upon probe data of DNA methylation to identify the characteristics of CAR‐T cells in a variety of B‐cell cancers [91].

Another study provided large‐scale methylation datasets and exhaustion‐associated methylation signatures that AI models can exploit. By mapping genome‐wide methylation dynamics of CAR‐T cells pre‐ and postinfusion, the study created a high‐resolution atlas of epigenetic states linked to therapeutic response and T cell exhaustion [92].

The field of CAR‐T cell detection, which still depends on human effort to distinguish the existence of CAR‐T cells following therapy, has benefited from another recent work. An expert blood morphologist labeled hundreds of CAR‐T cell images that were successfully assembled into a CAR‐T dataset for this investigation. In this study, a deep learning model was developed to classify the CAR‐T cell dataset. The model can assist the physicians in the detection of CAR‐T cells, which is one of the most important elements for clinical decision‐making of acute leukemia treatment [93].

A different study created a library of CARs with synthetic costimulatory domains made by combining signaling motif combinations. Different human T cell fates were encouraged by these CARs, and these fates were dependent on the arrangements and combinations of motif. Key design criteria were extracted by using trained neural networks for decoding the CAR signaling motifs combinatorial grammar.

In order to generate a CAR costimulatory domain library with randomized motifs combination, this study recombined signaling motifs. A variety of symptoms were developed using this library. It employed AI for signaling motifs language decoding, and extract design principles that guide the engineering domains of CAR signaling [94].

### 4.2 Predictive Modeling for Clinical Outcomes: Toxicity, Efficacy, and Resistance

From a therapeutic perspective, effective prognostic predictive models might be constructed by combining biomarkers linked to the response of CAR‐T cells with AI. One difficulty is that employing AI to create robust models necessitates the compilation of big datasets; thus, in order to prevent overfitting, data from multiple institutions must be combined. More complex factors, such as tumor mutational burden [95, 96, 97, 98], changes in antigen‐presenting pathways [69, 99], tumor microenvironment [100], downregulation or loss of tumor antigen [57, 59], and senescent phenotype [101, 102], can be analyzed with AI. The right qualification for therapy, relapse risk, response prediction, and timing (early vs. late relapse) could all benefit from these data [99, 101, 102]. A variety of straightforward variables, including lactate dehydrogenase (LDH), platelet (PLT) count, C‐reactive proteins (CRP), and performance status, have been shown to be significant in predicting responses to CAR‐T cell treatment [66].

#### 4.2.1 MicroRNA Prediction

These days, tumor neoantigens of individual patients can be identified and screened due to the advancements in genome sequencing technology, MHC epitope databases, and prediction algorithms. One of the most pressing issues in contemporary immunology is the inability to predict the precise binding of tumor neoplasm antigen to T cells due to the intricacy of T cells and the unpredictability of tumor variation. Traditional techniques including the tetramer assay and MHC tetramer assay are expensive, time‐consuming, and technically difficult [103]. A study group produced a deep learning model that offers a platform prediction approach and saves researchers costly and time‐consuming experiments due to the high‐speed computing benefits of AI and machine learning. Three primary data points were used in the training and prediction of microRNA, which is thought to be involved in the nervous system: the antigen sequence of T cell–tumor cell binding, the major tissue‐compatible complex (MHC) sequence, and the TCR sequence [104].

#### 4.2.2 Severe Cytokine Release Syndrome Prediction

Another field that benefits from AI is the prediction of the incidence and development of severe cytokine release syndrome (sCRS) which has been a fatal condition that is caused by CAR‐T cell therapies and can be of great impact. AI research on current healthcare data related to sCRS prediction could yield insightful findings.

A decision tree method was used in one of the most recent researches to forecast when severe CRS will strike adults and children. It was possible to predict with accuracy that patients would experience severe CRS using the decision tree model, which was based on three clinical factors including initial value prediction and prediction of same‐day and day‐ahead. Serum biomarker changes, such as those in ferritin and CRP, were linked to CRS but not on their own indicate the onset of severe CRS.

Furthermore, it was found that a classification model with high specificity and sensitivity would be able to predict if the patient is liable to severe CRS based on the patient's preliminary cytokine levels during the first phases of treatments [105] [106] [107].

This study has examined and determined the clinical characteristics associated with severe CRS as well as biomarkers, which accurately predict an onset of severe CRS. With the development of those models, patients with sCRS would be able to begin receiving active support treatment and be regularly monitored. In addition, anticipating the possible onset of sCRS will cause avoidance of unrequired cytokine treatment. This model has direct clinical and therapeutic value; it was developed based on the prediction of sCRS utilizing AI [108].

#### 4.2.3 Severe Immune Effector Cell‐Associated Neurotoxicity Syndrome Prediction

Accurately anticipating onset of severe ICANS following CAR‐T cell treatment is considered a vital process for most of the patients. Researchers have shown that AI and machine learning can accurately predict an onset of severe ICANS from clinical laboratories and vital signs data of patients receiving the standard‐of‐care CAR‐T cell therapies for B‐cell lymphomas. XGBoost, a popular supervised machine learning technique that enables highly flexible modeling by training decision trees ensembles to learn repeatedly from previous trees, was used to generate the predictions. To evaluate calibration and overfitting, K‐fold cross‐validation was employed. Age, CRP, ferritin, IL‐6, LDH, PLT counts, fibrinogen, and temperature were among the variables evaluated at preinfusion, Days 0 and 3 postinfusion time points. Temperature, age, and easily available laboratories data, such as CRP, ferritin, fibrinogen, IL‐6, LDH, and PLT counts, were all included in the model, which accurately predicted severe ICANS in patients receiving routine CAR‐T cell therapy before symptoms appeared. This makes it possible to early identify the patients who can most expected to experience severe ICANS and could benefit from preventative measures [109].

#### 4.2.4 Treatments Outcome Prediction in B‐Cell Lymphomas

The viability of using rule‐based reasoning and methodologies of deep learning‐based images analysis to predict therapeutic responses to CAR‐T cells therapy in B‐cells lymphomas was tested using computed tomographies, fluorodeoxyglucose positron emission tomographies and low‐dose CT images. Using these independent images, a pretrained neural networks model was utilized to forecast lesion‐level therapy responses. Lesion‐level prediction results were subjected to rule‐based reasoning in order to undertake patient‐level response analysis. When making decisions before starting CAR‐T cell therapy, this method may offer therapeutically valuable prognostic data.

Prediction of lesion‐level response was carried out by encircling each of abnormal lymph node in a 3D space with a rectangular box utilizing CAVASS software through utilization of volume of interest (VOI)‐based and entire slice‐based (non‐VOI) techniques [110].

This study showed that rule‐based reasoning prediction for patient‐level response performed better than prediction based on clinical risk variables. Additionally, it showed that a new image analysis by a deep learning method based on PET/CT and CT images may be used to effectively predict lesion‐level responses for patients who receive CAR‐T cell therapy for lymphomas. It also showed that it is possible to forecast patient outcomes with accuracy utilizing a rule‐based reasoning technique. According to the study's findings, these methods might offer innovative data that can be utilized to anticipate which individuals will benefit from therapy before it is started [111].

#### 4.2.5 Prediction of Action of CAR‐T Cells Against Severe Acute Respiratory Syndrome Coronavirus 2

Some studies employed kinetic models, branching processes, and Moran processes as modeling techniques that drew inspiration from the probabilistic laws. In the domains of data science and AI, the Moran processes are widely known. The infectious axis, “virus–CAR‐T cells–memory cells,” is shown in the model. Theoretical study suggests a beneficial therapeutic activity; early infection stages may be significantly impacted by the delay in viral generation. Even so, it was important to thoroughly consider any potential negative consequences of treatment. This can raise the prospect that CAR‐T cells could be used as an antiviral approach.

The effect of CAR‐T cells against severe acute respiratory syndrome coronavirus 2 (SARS‐CoV‐2)‐infected cells was predicted in a study. A few mappings were created, such as the kinetic modeling and the Moran process. The different distinct Moran type process trajectories that indicated the quantity of N type proteins in cells populations were created using a MATLAB method. The model used in this work was predicated on the idea that CAR‐T cells may be used to deliver antiviral treatment against SARS‐CoV‐2. This theory was confirmed by the quantifiable outcomes that AI produced [112].

#### 4.2.6 Tumor Neoantigen Prediction Models

According to Calis et al., neopeptide–MHC combinations have two characteristics that lead to variations in the recognition of T cell. The first characteristic is amino acids compositions in the location of the existing peptides. The second characteristic is the extent and presence or absence of aromatic side chains [113]. The aforementioned characteristics are among the nine immunogenicity factors integrated by Neopepsee, a machine‐learning‐based neoantigen prediction algorithm that was able to identify melanoma immunogenic neoantigens. Prediction models that raises the precision of neoantigen prediction, like bioinformatics tool for loss of heterozygosity in human leukocyte antigen (HLA), allow from data sequencing to estimate loss of allele‐specific HLA [68, 114]. A significant disadvantage of tumor neoantigen prediction systems continues to be their high false positive rate [115]. Nevertheless, the implementation of these prediction models in clinical practice will require prospective validation and replication in an actual environment.

### 4.3 Enhancing Manufacturing and Potency Control Through Process Analytics

The area of CAR‐T cell therapy is suffering from absence of comprehensive insight into the bioprocesses and complicated labor‐intensive manufacturing. Because of the high manufacturing costs and poor clinical outcomes, CAR‐T cell treatments are not widely used. In order to raise manufacturing capacity and decrease provision times, new manufacturing techniques are required to reduce costs and efforts.

Hospitals and treatment facilities face additional difficulties as a consequence of the production and distribution of CAR‐T cells. Because the therapy is autologous, hospitalized patients’ T cells are extracted, sent to pharmaceutical companies or academic institution to manufacture CAR‐T cells, and then given back to the patient to be infused.

Recent studies provided insights into soft sensors of the state of the art and AI for cells cultures control. One survey highlighted automated cells expansion trends and key performance indicators (KPIs) including artificial neural networks, cell count, foaming, glycosylation, viability, morphology, biomass, Raman spectroscopy, highlighting fluorescence, and chemometrics. The other study carried out an extensive investigation including a variety of contemporary sensor technologies, such as artificial neural network, optical sensor, spectroscopy, wireless sensors that are free floating, and statistical techniques for antibody titers and cells density modeling [78, 79].

The development of the CAR genes and its impact on cell and tumors before production is a further area receiving a lot of attention. Dannenfelser et al. studied associations between several potential indicators and their impacts on tumor cells, and attempts were made to forecast potential efficacy [116].

A study project looked into additional use cases for AI in the production of CAR‐T cells in addition to the ones listed in the literature. The expansion of the CAR‐T cells into bioreactors is directly related to two of those use cases. The first use case was to create a digital twin for the bioreactor through modeling its design and operation mechanistically. It also involved modeling the growth of CAR‐T cells by simulating their intake of essential nutrients and generation of metabolites. This digital twin offered both short‐term projections of future cell concentration and a soft sensor of future cell concentration. Based on an evaluation of whether the target dose which is the minimum number of cells needed for treatment has been met, these forecasts can then be used to determine when to end the expansion phase. For bioreactor parameters real‐time monitoring, a reactive online process that is based on a set of soft sensors control was created in the second use case. Various soft sensor algorithms, such as AI and statistical methods, increased the level of trust in evaluating the overall circumstances [117, 118].

The CAR‐T cell product's ability to be personalized is another factor taken into account in this research. Theoretically, different patients need customized product attributes. These are weighed against competing hazards, such as the survival after therapy and the absence of tumors. Furthermore, the complementary therapy needs to be customized for the patient. A clinical decision support system could help in this situation [119]. A mathematical model by machine learning was proposed in two stages. The first stage consists of development and validation of prediction models through the use of historical datasets. In the second stage, these models will be implemented on new patients for deciding the most effective in survival optimization for each patient. This will allow for personalized infusion and production of optimal CAR‐T cells [76].

By following the product through the whole production process and simulating cell behavior, a digital twin can provide greater insights into the CAR‐T cell process and how it can be affected by patient variables affect it. The control software can adjust the bioreactor during the prolonged cells expansion processes based on this observation. The bioreactor's recorded data of the process, can be used to simulate potential expansion strategies and assess the condition of the cells [120, 121, 122, 123]. Because metabolomics data have promising qualities for quality control in individualized therapy, they are added to the process data [124, 125]. AI can be used to plan CAR‐T cell therapy by resolving a difficult resource allocation problem with significant uncertainty [126, 127]. Variations in manufacturing schedules and the quantity of resources required, like medical devices or intensive care unit beds, are the sources of the complexity. In addition, based on each patient's unique progress, the duration of therapy can be modified during sessions [127, 128]. While traditional optimization algorithms have reached their limits, reinforcement learning appears to be a potential approach that can overcome these obstacles. Also, the adaptive scheduling can best integrate the platform's manufacturing processes with the therapy process as a whole [127, 129].

Comprehensive understanding of the hardware components such as machines and devices and software components such as AI models, data management, and control software is necessary for the establishment of manufacturing platform of CAR‐T cells, which is automated and AI driven [80]. The integration of all necessary hardware, including machines and devices, into a single integrated process pipeline is required to automate the manufacturing of autologous CAR‐T cell treatment.

An automated tubing‐kit‐based devices was used by a previous study in which CAR‐T cell manufacturing has been achieved. Through joining tube assemblies, cells are immediately moved to the next stage of their processing. The establishment of an integrated perfusion bioreactor can be considered a key element in the manufacturing process as it is designed with several sensors for the deployment of the AI‐supported control approaches; moreover, it can enable cocultivation of CAR‐T cells as much as it is required. Integrating automation enabled solutions for routine laboratory operations completes the quality control sector [80]. The manufacturing of CAR‐T cells is a costly procedure due to the materials needed as well as the labor‐intensive nature of the process.

### 4.4 Deciphering Complex Biology: AI in Modeling Trogocytosis and Tumor–Immune Interactions

Deciphering the complex rules governing trogocytosis and other multifaceted tumor–immune interactions can be beyond the capacity of regular experimental approaches alone. AI can offer a transformative lens for trogocytosis. By applying machine learning to high‐dimensional single‐cell data, live imaging, and spatial transcriptomics, AI can assume the precise molecular and cellular conditions that precipitate trogocytosis and predict its clinical consequences [130, 131].

Some studies used a high‐dimensional reduction algorithm for detection of NK cell subsets, which degranulated after active trogocytosis performance [81, 82].

To achieve further detection of the tumor markers expression presented on surfaces of NK cells because of trogocytosis, an algorithm for detecting of the t‐distributed stochastic neighbor embedding was used. The results showed marker expression differences in several spatial regions suggesting presence of NK cells subsets with altered expression and abundance of these markers [83].

For trogosome material tracking in microscopy, a 3D fast tracking algorithm was developed. A preliminary requirement for utilization of this algorithm is the precise detection of point sources in the 3D microscopy data of the whole cell. This algorithm performance has been tested with real data [84].

### 4.5 Modeling and Simulations for Examining Various Features of CAR‐T cell Therapy

Models and simulations that use AI can determine the variables affecting the safety and efficiency of nonuniform CAR‐T cell treatment. Moreover, these models may also be able to determine patients with good prognosis or bad prognosis. This could allow for the planning of risk mitigation strategies, which could lower the mortality rate and complications associated with CRS symptoms [67].

Various models have been created to examine various features of CAR‐T cell therapy, such as machine learning models for determining CRS biomarkers [105]; examine the interactions that occur between cytokines, tumor cells, and CAR‐T cells [132, 133]; study effects of tocilizumab [134]; understand CAR activation mechanisms by using models at the cellular level [135, 136]; clarify the temporal interactions between memory T cells, CAR‐T cells, and cancer cells using in vivo data to gain a better comprehension of the relative effects of tumor immunosuppressive environments, cell death, and proliferation of CAR‐T cell therapy [72, 73, 74]; describe glioma and CAR‐T cells temporal interplay using patient data from in vivo experiments and in vitro testing [73]; clarify how CAR‐T cells and cancerous CD19+ B cells interact [137, 138]; demonstrate the factors affecting therapeutic outcomes [139]; describe how malignant CD19+ B cell, effector CAR‐T cell, memory CAR‐T cell, and normal memory T cells interact to better comprehend the relative effects of malignant cell death and proliferation on the overall effectiveness of CAR‐T therapy [140]; analyze the correlation between treatment results and product attributes by the application of regression and classification tree clustering [141]; determine the dosage and patient circumstances that would yield the best possible therapeutic response [142]; and assess CAR‐T cell therapy cost effectiveness [143, 144, 145, 146].

**FIGURE 4:**
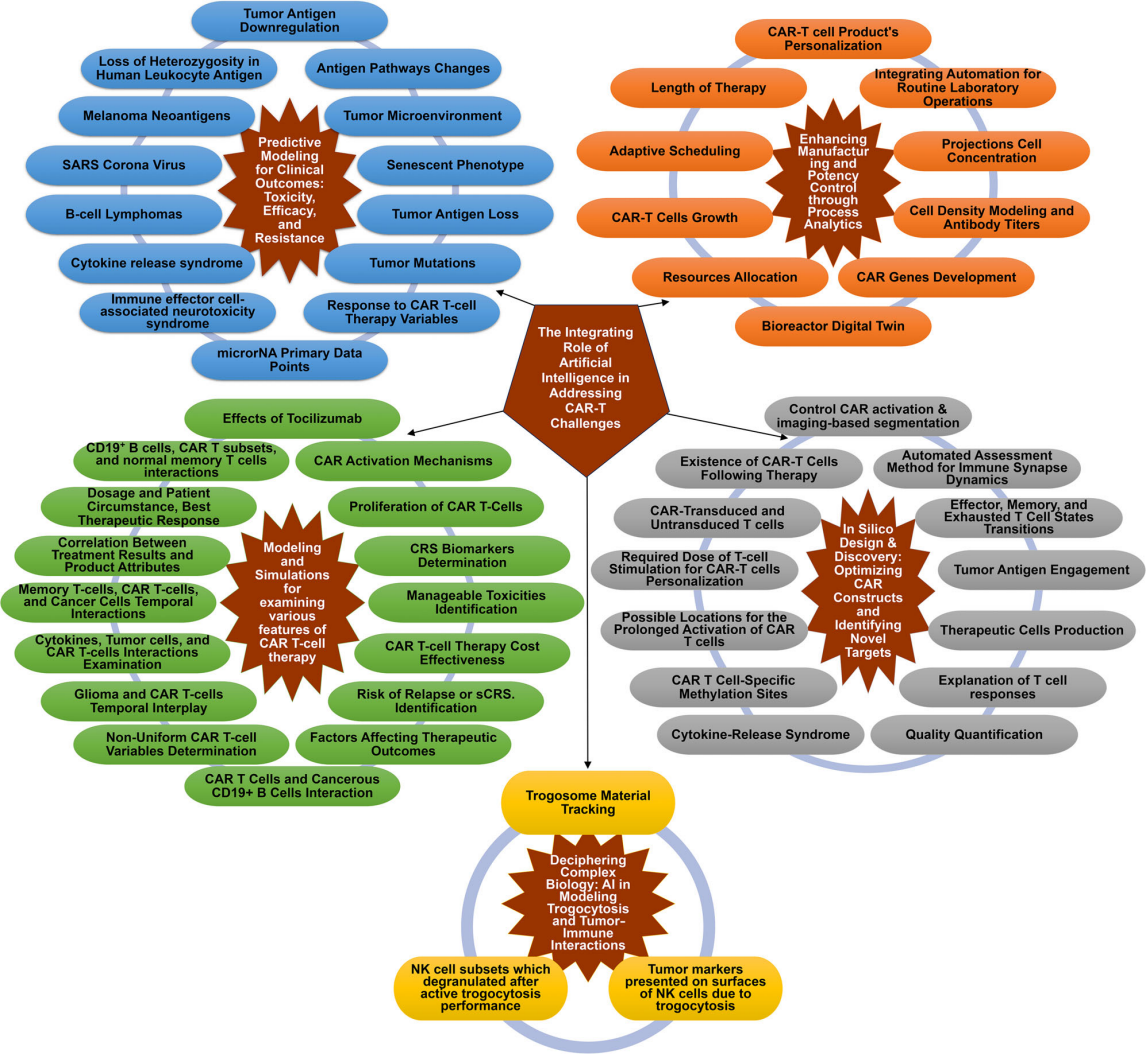
Summary of the role of AI in CAR‐T cell therapies that were categorized into five areas of research including optimization of CAR constructs, predictive modeling, manufacturing enhancement, and deciphering complex biology.

These proposed mathematical simulation models can reduce time and costs through identification of the underlying causes of therapeutic failure and may also lead to improved clinical protocols. To conclude, Table 2 presents the current AI applications that have been integrated for addressing CAR‐T cell therapy as well as the suggested methods, roles, mechanisms, and outcomes for each application. Figure 4 presents a summary of the integrating role of AI in CAR‐T cell therapies.

**TABLE 2:** AI applications, suggested methods, roles, mechanisms, and outcomes for addressing CAR‐T cell therapy.

Application	AI suggested method	AI role	Mechanism	Outcomes	References
**In silico design and discovery: optimizing CAR constructs and identifying novel targets**					
Control CAR activation and imaging‐based segmentation	Deep learning, reinforcement learning	Process optimization	Deep learning‐based segmentation, reinforcement learning	Improved CAR‐T efficiency	[70, 87]
Automated effectiveness assessment method for immune synapse dynamics	Machine learning‐based scoring	Synapse quality assessment	Image, machine learning classification of synapse	Correlation with patient outcomes	[58, 87]
Transitions between effector, memory, and exhausted T cell states	Machine learning trajectory models	State transition modeling	Model dynamic differentiation	Optimize long‐term CAR function	[88]
Tumor antigen engagement	Deep learning classifiers	Engagement modeling	Quantify antigen recognition patterns	Better prediction of efficacy	[88]
Therapeutic cells production	Reinforcement learning optimization	Manufacturing enhancement	Optimize expansion control	Scalable CAR‐T production	[70]
Explanation of T cell responses	Deep learning, regression	Response deconvolution	Decompose patient variance in response	Improve clinical predictability	[88]
Quality quantification	Machine learning‐based quantification	CAR‐T quality control	Measure synapse quality with machine learning	Standardized potency assays	[86]
Cytokine‐release syndrome	Machine learning classifiers, random forest, regression modeling	Early detection	Use patient cytokine profiles to identify CRS biomarkers	Predict severe CRS risk before onset	[105, 106, 107]
CAR‐T cell‐specific methylation sites	Machine learning classifiers	Epigenetic signature detection	Identify DNA methylation patterns unique to CAR‐T	Monitor T cell exhaustion and persistence	[91]
Possible locations for the prolonged activation of CAR‐T cells	Spatial machine learning models	Functional mapping	Link T cell activity to spatial tumor niches	Predict durable response zones	[90, 91]
Required dose of T cell stimulation for personalization of CAR‐T cells production	Regression machine learning models	Dose personalization	Learn dose–response from patient data	Safer personalized therapy	[75]
CAR‐transduced and untransduced T cells	Deep learning classifiers	Cell population discrimination	Use single‐cell omics for classification	Accurate identification of CAR+ T cells	[91]
Existence of CAR‐T cells following therapy	Deep learning model (RCMNet)	Persistence tracking	Model circulating CAR‐T in blood	Monitor therapy durability	[93]
**Predictive modeling for clinical outcomes: toxicity, efficacy, and resistance**					
Tumor antigen downregulation	Predictive machine learning models	Resistance prediction	Simulate antigen escape patterns	Anticipate relapse	[59, 66]
Antigen pathways changes	Pathway machine learning analysis	Mechanism prediction	Detect altered signaling and antigen processing	Optimize CAR targeting	[58, 86]
Tumor microenvironment	Graph learning, deep learning	Microenvironment modeling	Decode immune‐suppressive niches	Predict therapeutic resistance	[100]
Senescent phenotype	Feature‐based machine learning	Senescence detection	Nuclear and transcriptomic features	Identify senolytic targets	[101, 102]
Tumor antigen loss	Hybrid machine learning models	Antigen escape detection	Integrate antigen tracking with outcome models	Better re‐targeting strategies	[58, 59]
Tumor mutations	Rule‐based and machine learning approaches for antigen prioritization (NeoDisc pipeline), deep learning, decision trees	Neoantigen prediction	Multiomics mutation profiling	Personalized target discovery	[95, 96, 97]
Response to CAR‐T cell therapy variables	Machine learning regression	Response prediction	Learn associations between biomarkers and response	Personalized therapy	[66]
microrNA primary data points	Machine learning, deep learning	Feature extraction	Identify microRNAs predictive of CAR‐T efficacy	Biomarker‐guided therapy	[104]
Immune effector cell‐associated neurotoxicity syndrome	Gradient‐boosted trees	Early toxicity prediction	Analyze clinical and lab variables	Safer patient monitoring	[109]
Cytokine release syndrome	Random forest, deep learning	Toxicity prediction	Predict CRS severity from cytokine trends	Prevent life‐threatening CRS	[105, 106, 107]
B‐cell lymphomas	Deep learning imaging	Response prediction	Tumor imaging, machine learning analysis	Stratified therapy prediction	[111]
SARS corona virus	Stochastic machine learning models	Antiviral response prediction	Model CAR‐T vs. virus interaction	Potential CAR‐T antiviral role	[112]
Melanoma neoantigens	Machine learning neoantigen models	Antigen prediction	Predict immunogenic peptides	Improved target design	[114]
Loss of heterozygosity in human leukocyte antigen	Machine learning, deep learning	Immune escape prediction	Detect allele‐specific HLA loss	Predict resistance	[115]
**Enhancing manufacturing and potency control through process analytics**					
CAR‐T cell product's personalization	Machine learning, deep learning, unsupervised learning	Patient‐specific improvement	Analyze omics, biomarkers, and prior therapy data to design tailored CAR constructs	Improved therapeutic efficacy and reduced relapse	[75, 76, 119]
Integrating automation for routine laboratory operations	Neural networks, robotics integration	Process automation	Automate quality control, cell expansion, and monitoring	Reduced human error, faster turnaround	[80]
Projections cell concentration	Machine learning models	Controlling	Use time‐series and sensor data to project viable cell concentrations	Improved culture time and yield	[117]
Cell density modeling and antibody titers	Machine learning regression, artificial neural networks	Process modeling	Correlate density metrics with antibody expression	Better culture productivity	[79]
CAR genes development	Generative deep learning	Target design	Envisage optimal CAR gene constructs with multiantigen recognition	Enhanced tumor specificity, reduced off‐target effects	[116]
Bioreactor digital twin	Digital twin, machine learning	Process enhancement	Mirror patient‐specific manufacturing conditions digitally	Safer predictions of cytokine release and toxicity	[117, 120]
Resources allocation	Reinforcement learning	Decision optimization	Reinforcement learning allocates bioreactor, media, and workforce dynamically	Efficient resource use, cost reduction	[126, 127]
CAR‐T cells growth	Multimodal deep learning	Growth prediction	Integrate omics and metabolic markers for dynamic growth models	Improved expansion efficiency	[117, 118]
Adaptive scheduling	Reinforcement learning, scheduling algorithms	Workflow management	Adjust batch scheduling in real time to demand and constraints	Reduced waiting time, better throughput	[127, 129]
Length of therapy	Mechanistic, machine learning hybrid models	Therapy outcome modeling	Predict persistence and expansion kinetics in vivo	Optimized dosing and therapy duration	[127, 128]
**Deciphering complex biology: AI in modeling trogocytosis and tumor–immune interactions**					
Trogosome material tracking	Image‐based calculations, regression‐based model, segmentation	Cell interaction tracking	Detect transfer of membrane fragments between cells	Better understanding of trogocytosis in CAR‐T	[84]
Tumor markers presented on surfaces of NK cells due to trogocytosis	Unsupervised neural network (FlowSOM), machine learning pipeline (CITRUS)	Antigen detection, feature extraction	Detect tumor antigens acquired by NK cells	Early relapse marker	[83]
NK cell subsets that degranulated after active trogocytosis performance	Unsupervised machine learning (UMAP), machine learning clustering	Subset identification	Analyze degranulation and phenotypic changes	Better immune monitoring	[81, 82]
**Modeling and simulations for examining various features of CAR‐T cell therapy**					
Effects of tocilizumab	Machine learning pharmacokinetic models	Toxicity mitigation modeling	Simulate IL‐6 blockade	Better CRS management	[134]
CAR activation mechanisms	Computational machine learning, deep learning	Mechanistic modeling	Model phosphorylation and ERK activation	Mechanistic understanding	[135, 136]
Proliferation of CAR‐T Cells	Kinetic models, machine learning	Expansion modeling	Multicompartment PK‐PD	Predict expansion	[72, 73, 74]
CRS biomarkers determination	Machine learning classifiers	Toxicity marker detection	Integrate cytokine profiles	CRS early warning	[105]
Manageable toxicities identification	Simulation, machine learning	Toxicity prediction	Model patient‐specific toxicity	Personalized safety	[67]
CAR‐T cell therapy cost effectiveness	Machine learning cost models	Economic optimization	Predict outcomes vs. costs	Policy and reimbursement guidance	[143, 144, 145, 146]
Risk of relapse or sCRS identification	Hybrid machine learning	Relapse prediction	Integrate patient and biomarker data	Risk stratification	[67, 99, 101, 102]
Factors affecting therapeutic outcomes	Systems machine learning	Multiscale prediction	Model CAR affinity, antigen load	PK–PD optimization	[139]
CAR‐T cells and cancerous CD19+ B cells interaction	Simulation, machine learning	Immune–tumor modeling	Simulate CAR‐T and B cell killing	Better dosing guidance	[137, 138]
Nonuniform CAR‐T cell variables determination	Machine learning clustering	Variability modeling	Capture heterogeneity in CAR‐T kinetics	Refined patient stratification	[67]
Glioma and CAR‐T cells temporal interplay	Dynamic simulation	Interaction modeling	Simulate tumor and CAR kinetics	Better tumor targeting	[73]
Cytokines, tumor cells, and CAR‐T cells interactions examination	ODE models, machine learning	Multiscale simulation	Integrate immune–tumor–cytokine feedback	Predict CRS and efficacy	[132, 133]
Memory T cells, CAR‐T cells, and cancer cells temporal interactions	Compartmental machine learning models	Dynamics simulation	Model long‐term persistence	Predict durability	[72, 73, 74]
Correlation between treatment results and product attributes	Machine learning regression	Correlation modeling	Link CAR‐T attributes to patient outcomes	QC‐based therapy optimization	[142]
Dosage and patient circumstances yielding best possible therapeutic response	Optimization machine learning	Dosing personalization	Identify best dose per patient	Better safety‐efficacy	[142]
Malignant CD19+ B cells, memory CAR‐T cells, effector CAR‐T cells, and normal memory T cells interaction	Stochastic machine learning models	Immune dynamics	Model interaction between malignant and CAR/memory T cells	Explain therapy success/failure	[140, 147]

Abbreviations: CITRUS, cluster identification, characterization, and regression; ERK, extracellular signal‐regulated kinase; FlowSOM, flow cytometry self‐organizing maps; IL‐6, interleukin‐6; NeoDisc, neoantigen discovery; PK‐PD, pharmacokinetics–pharmacodynamics; RCMNet, regression‐based confidence map. Prediction network; UMAP, uniform manifold approximation and projection.

AI implementation in the CAR‐T cell therapies has created a robust conceptual background and the real challenge to these advances is to apply them in the real‐life context [148, 149]. Theoretical and computational advances must ultimately be validated through experimental models and clinical trials, where the power of AI can be measured against patient outcomes and therapeutic safety [150, 151]. This natural advancement from in silico investigation to in vivo and clinical assessment demonstrates the necessity of bridging discovery with application, preparing an analysis of experimental and clinical AI trials within CAR‐T cell therapies.

## 5 From Bench to Bedside: Clinical Translation and Ongoing AI‐Assisted Trials

Bridging long‐standing gaps between basic science and clinical practice is revolutionizing the translational research of AI [152]. Several successful cases of bench‐to‐bedside translation has been demonstrated in the literature [153].

### 5.1 Review of Key Clinical Trials Incorporating AI Elements

Machine learning and deep learning models have been employed to link preclinical features with patient outcomes, design novel antigen binders, and analyze imaging data for response prediction [66, 154]. In parallel, interpretable models and multimodal frameworks have been developed to identify early biomarkers of CRS, stratify patient risk, and forecast severe toxicities several days in advance [155]. Digital twin simulations further extend these efforts by modeling systemic inflammatory reactions and assisting bedside decision making [156]. All together, these AI‐driven approaches improve accuracy in patient selection, monitoring, and management, thereby improving safety and treatment outcomes of the clinical deployment of CAR‐T cells. Table 3 presents the experimental and clinical studies that incorporated AI in CAR‐T cells.

**TABLE 3:** Experimental and clinical studies using AI in CAR‐T cell therapy.

Experimental and clinical trials	AI method	AI role	Mechanism	Outcomes	References
Linking preclinical models to patient outcomes	Guided regression model machine learning analysis linking mouse model features to clinical outcomes	Detect preclinical features that predict clinical efficacy	Standard CAR‑T cytotoxic pathways (perforin–granzyme; death‐receptor) linked to in vivo tumor control features	Preclinical features sometimes predict clinical outcomes.	[154]
Design of novel antigen sensors for CAR‐T	Generative models that rapidly create specific binders to pMHC	Manufacture new antigen sensors for engineered T cells	T cell cytotoxicity through the AI‐designed pMHC binders that enable antigen recognition	High‐affinity binders	[157]
Radiological prediction of CAR‐T response	Deep learning‐based image analysis and rule‐based reasoning Transfer learning via pretrained neural network models	Predict lesion‐level treatment response to CAR‐T cell therapy from separate radiological images	CAR‐T cytotoxicity and persistence linked to imaging correlates of tumor burden/biology	High patient‐response level accuracy Outperformed international prognostic index	[111]
CRS prediction (timing and severity)	Deep learning prediction model based on U‐nets and transformers	Predict both the timing and likelihood of severe CRS in patients	Cytokine‐driven inflammation and macrophage activation cascades	Best performance among comparators in prediction with 1, 2, and 3 days in advance Open‐source code	[158]
Early CRS biomarker detection	Interpretable machine learning (decision tree), an early predictive model	Detect early biomarkers that are associated with severe CRS	Cytokine signaling and inflammatory biomarkers	Good sensitivity in prediction of severe CRS after CAR‐T cells infusion, incorporating readily accessible clinical parameters	[106]
Early CRS risk stratification	Machine learning model leveraging multimodal patient characteristics	Predict if/when CRS will occur (lead time up to 3 days)	Inflammatory cytokine signaling underlying CRS	Reported reliable early risk stratification	[107]
Digital twin for CRS management	Digital twin simulation integrating clinical data	Model CRS grade distributions with decision support	Systemic inflammatory response modeling	Feasibility proof for digital‐twin approach	[120]
Preinfusion response prediction (multimodal)	Machine learning multimodal framework model	Predict preinfusion response	Integrative biomarkers attached to CAR‐T cytotoxic pathways	Multimodal models significantly improved the prediction of complete response compared with individual modalities.	[159]
Preinfusion risk stratification via proteomics	Machine learning on plasma protein signatures	Predict preinfusion outcome	Inflammatory/proteomic pathways associated with tumor clearance	Identified high‐risk groups for relapse/mortality	[119]

### 5.2 Case Studies: AI in Predicting and Managing CAR‐T‐Related Toxicities

According to a study by Wang et al., in the multicenter University of California, a pivotal application of AI in managing CAR‐T cells was demonstrated by correlating early biomarker patterns to toxicity risks. The authors identified that lower postinfusion levels of certain biomarkers were significantly associated with the development of CRS and ICANS. While the primary machine learning model was designed to predict early relapse, its decision tree structure is directly implicated in toxicity. This generates a dual‐purpose tool, as the model not only stratifies relapse risk but also detects patients with a physiological profile indicative of severe toxicities, allowing preemptive clinical management [149].

When compared with traditional methods, a retrospective case analysis demonstrated that a multiagent AI system could accurately detect toxicity risks for targets including Fc Receptor Homolog 5 (FcRH5) and CD229, as well as offer deeper mechanistic insights and more useful risk‐mitigation strategies.

This clinical translation establishes that a collaborative, multiagent framework can effectively address central inefficiencies in CAR‐T development by integration of the processes of safety prediction, target discovery, and molecular design [160]. Therefore, AI can synthesize disparate data sources to proactively predict and help manage serious CAR‐T‐related toxicities.

## 6 Current Limitations and Hurdles of AI in CAR‐T Cell Therapy

AI holds tremendous promise for improving CAR‐T cell therapies, yet, its application is limited by several issues that should be taken into consideration before widespread clinical integration.

### 6.1 Data Quality, Availability, and Standardization

The validation of a reliable functioning of the AI algorithms and the assurance of its reliability are strongly dependent on the appropriateness of data quality for training as well as the possibility for continuous training [80]. Therefore, any bias can ultimately affect the critical quality attributes of the final outcomes [161]. Moreover, the lack of electronic medical records availability in the studies databases, can unable the validation of the studies algorithms [162]. The literature also revealed that the establishment of a standardized manner for reporting data on CAR‐T cell expansion/detection and persistence is clearly needed [163].

Data sparsity in rare cancer types presents an additional barrier. CAR‐T therapies are often trialed in hematological malignancies, but datasets for rarer cancers—and especially for solid tumors—remain too small to support robust AI training. This scarcity hinders the ability of algorithms to capture diverse biological variations, slowing advancement of CAR‐T applicability [164, 165]. Model overfitting is one of the foremost limitations in applying AI to CAR‐T therapy, where algorithms trained on small or highly specific datasets may capture noise rather than true biological patterns. This often leads to excellent performance in internal validation but poor results when exposed to new patient data, reducing clinical reliability [166].

Moreover, there is the critical issue of the need for prospective validation. While retrospective analyses and simulation studies demonstrate AI's potential, true clinical utility requires prospective, multicenter trials that test AI‐driven recommendations in real time [167, 168]. Without such validation, AI remains a promising adjunct rather than a trusted clinical decision tool.

Integrating AI insights into dynamic and multifactorial biological processes —such as trogocytosis, antigen escape, and cytokine signaling— is further complicated by the complexity of tumor microenvironments, which remain difficult to capture accurately in silico [71, 169].

### 6.2 Model Interpretability and “Black Box” Problem

Many AI approaches function as “black boxes,” offering predictions without clear mechanistic explanations, which limits their clinical trustworthiness in high‐stakes settings like oncology. When AI models are used to forecast patient outcomes, toxicity risks, or relapse probability, the “black box” nature of many machine learning approaches raises questions about accountability, transparency, and bias. If models inadvertently reflect systemic inequities in healthcare data, their deployment could worsen disparities rather than improve care [170, 171].

### 6.3 Regulatory and Ethical Considerations

The deployment of AI into CAR‐T cell therapy is severely restricted by a set of ethical limitations pertaining to data governance and security. The generation of robust AI models is predicated on access to large‐scale and heterogeneous datasets; however, this requirement is impeded by the lack of standardized data‐sharing frameworks and the existence of complex regulatory regimes. This setting can exacerbate major cybersecurity vulnerabilities inherent in the increasing digitization of healthcare, elevating the risk of data malware attacks on medical records of patients. Moreover, the ability for reidentification of deanonymized data by AI can deeply compromise the integrity and confidentiality of data.

Furthermore, the lack of societal knowledge and confidence about the usage of personal health information for AI research presents a significant socio‐ethical obstacle, potentially reducing participation, biasing datasets, and ultimately hindering the equitable and secure translation of AI‐driven innovations in CAR‐T treatment [117, 172]. These limitations highlight the importance of transparent, biologically informed, and clinically validated AI models in order to fully exploit their potential in CAR‐T therapy.

## 7 Future Perspectives

Advances in CAR‐T therapies offer new alternatives for treating refractory malignancies, and next‐generation CAR‐T clinical trials that are strategically designed to overcome various hematologic or solid cancers are ongoing [173]. AI is integrated to boost CAR‐T therapies capabilities by optimization of all phases, from targets selection, vectors design, and manufacturing to personalized data‐driven therapeutic decisions [18].

### 7.1 Next‐Generation CAR‐T Therapies Guided by AI Insights

More complex modeling and more sophisticated testing techniques would be needed to develop the next generation of CAR‐T cell treatments. AI and machine learning techniques are revolutionizing CAR‐T cell therapy by improving its efficacy and safety in the treatment of specific tumor types [150].

AI algorithms and machine learning models, introduced a revolution in several genome editing aspects. A study by Boretti et al. revealed that the integration of AI with CRISPR–Cas9 genome editing shows remarkable potential in the advancement of CAR‐T therapies. AI algorithms were able to offer unparalleled accuracy in identification of the genetic targets. This precision is considered essential for the elimination of negative regulatory elements that compromise therapeutic effectiveness [174].

Generative AI tools are optimizing the scFv, signaling domains, and spacer regions to improve tumors penetration, persistence, and activation [175].

In aggressive rare cancers like glioblastoma, AI techniques including predictive modeling and machine learning, are more integrated into multiscale models to improve the analysis of CAR‐T cells kinetics and dose–response relationship [176]. AI guided therapies are used to identify solid tumors biomarkers for refining CAR‐T cells designs and predict therapeutic responses [177].

The emergence of AI and machine learning has provided a promising avenue for introduction of CAR‐T cells in the treatment of lymphoma. By analyzing vast genomic and proteomic datasets, identification of optimal target antigens on lymphoma cells has become possible. These technologies can also reveal novel target antigens, which might not be readily apparent to human scientists [178].

AI is refining the entire CAR‐T pipeline. Computer vision enables real‐time monitoring of cell morphology during manufacturing. Reinforcement learning algorithms are being developed to optimize patient dosing regimens. Generative AI models are recently revolutionizing CAR design itself [175]. Furthermore, AI is used to anticipate major hindering challenges in the tumor microenvironment. These predictions directly inform the design of next‐generation CARs, equipping them to resist these obstacles and even reprogramming the hostile tumor microenvironment [179].

### 7.2 The Road Toward Personalized and Accessible CAR‐T Treatments

The central stone of the personalized CAR‐T cells paradigm is the introduction of the AI‐based tumor‐specific antigen identification. Large‐scale datasets from proteomics, transcriptomics, and cancer genomics can be integrated by AI models to find suitable targets that are poorly expressed in normal tissues but substantially expressed in cancers [180].

In clinical investigations, dose–response modeling has evolved to include patients’ specific covariates, allowing for more accurate treatment plans. A variety of techniques have been developed, including standard parametric models and semi‐parametric approaches that can address treatment effect heterogeneity [181].

Recently, CAR‐T cell personalization has been significantly improved by using Bayesian optimization, which takes patient characteristics and dose–covariate interactions into account [182].

It has been demonstrated that adding patient‐specific features, including binary covariates, greatly increases the accuracy of dose prediction and hence the value of personalized medicine in clinical investigations.

These advancements make it easier to tailor treatment plans for specific patient profiles, improving therapeutic efficacy and accuracy [183].

Both AI and machine learning have demonstrated revolutionary potential in enhancing the accessibility of CAR‐T cells through optimizing the production and reducing the costs [184]. Machine learning algorithms outperform traditional approaches in predicting patient outcomes based on preinfusion transcriptomes [185]. Furthermore, neural networks have the capability to form CAR constructs with appropriate signaling motifs, accelerating development and enhancing therapeutic accessibility [186].

The conventional methods of CAR‐T manufacturing rely on viral vectors, which have limited efficiency and may cause genotoxicity issues.

A study by Zhang et al. was able to increase CAR‐T manufacturing efficiency by 20‐fold through the adjustment of the osmolarity of the electroporation buffer [187]. According to this study, AI could be used to optimize buffer settings and estimate how they would affect intracellular signaling, thereby accentuating manufacturing consistency that improves accessibility [188].

### 7.3 Synergistic Integration of AI With Emerging Technologies

The synergistic integration of AI and Internet of things could enable for more personalization of CAR‐T cells, streamlining the therapeutic approaches [189]. This synergy can serve in prioritizing appropriate CAR targets through the interpretation of multiomics datasets such as spatial transcriptomics, proteomics, and RNA sequencing [190].

Incorporation of various omics technologies, single‐cell studies, and immunogenetics with bioinformatics is central for advancements of CAR‐T cell therapies. Bioinformatics is central for assessment of large‐scale genomic and proteomic data and uncovering new targets for better comprehension of complex cellular activities [191]. Furthermore, omics analysis and single‐cell technologies allow for the deep investigation of individual cellular behaviors, resulting in a high‐refined knowledge of the variability of CAR‐T cells. These interdisciplinary efforts are ushering a new era in improving the efficacy of CAR‐T cell therapies [190].

The development of biocompatible materials and advanced delivery mechanisms by biomedical engineering integration significantly augments the in vivo efficacy of therapeutic CAR‐T cells. Their clinical translation, however, is contingent upon innovations in scalable manufacturing processes capable of supporting personalized medicine. Future work should prioritize the creation of modular platforms designed for the continuous integration of emergent discoveries and patient‐specific data [189].

Fusion of AI with nanotechnology also represents a significant shift in confronting the barriers facing CAR‐T therapies. This combination pioneers a potent synergy between CAR‐T innovation and nanotechnology, denoting a transformative advancement in the field of precision oncology. As AI‐driven models enable personalized manufacturing and dynamic adjustments, nanotechnology on the other hand can facilitate safer, nonviral gene delivery, and precise, localized control over the tumor microenvironment. Together, they boost CAR‐T cell potency and persistence while minimizing systemic toxicity. Moreover, the intelligent CAR‐T nanotechnology extends the boundaries of existing methods by enabling the in vivo generation of CAR‐T cells. This approach uses AI‐optimized lipid nanoparticles for delivering CAR constructs directly into a patient T cells, eliminating the complex and expensive need for ex vivo manipulation [192].

## 8 Concluding Remarks

This review highlighted that CAR‐T cell mechanisms, including the emerging role of trogocytosis, provide the foundation for both therapeutic success and resistance. The core mechanisms of CAR‐T cell action are demonstrated in light of current research trends, with a focus on functional quality and postinfusion biology. Understanding the biological basis of CAR‐T cells, together with the development of intelligent solutions to address existing challenges can be considered as the elegant redirection for achieving therapeutic advances. The current challenges and limitations of CAR‐T therapy remain as a major clinical barrier. Results indicated that AI applications are increasingly being tailored to these barriers—such as simulations that can identify patients with manageable toxicities, analyze the changes in antigen‐presenting pathways, enhance the accuracy of neoantigen prediction, and analyze particular chemokine profiles, and tumor microenvironment. Furthermore, AI can improve personalization of CAR‐T cells production, enable cocultivation of CAR‐T cells as much as it is required, and track trogosome material.

Within this context, the review findings show that AI can model receptor–antigen interactions, predict cytokine signaling cascades, and anticipate exhaustion pathways. Moreover, in the context of trogocytosis, it can help map antigen transfer dynamics. However, the effectiveness of these computational strategies depends on robust biological validation.

The integration of AI into CAR‐T research is further supported by clinical trials, which demonstrate success in prediction, patient stratification, and therapy optimization. However, results also reveal that translation into clinical decision‐making is still limited, constrained by small datasets, lack of cross‐institutional reproducibility, and the absence of prospective validations.

The review also emphasizes the constraints that faces AI in CAR‐T therapy, which must be addressed before AI can be matured from exploratory applications into standardized tools for treatment.

In summary, comprehension of mechanistic biology of CAR‐T cells can significantly optimize the treatment outcomes. AI can potentially introduce reliable and novel paradigms for CAR‐T cells therapy services. Moreover, there is a necessity for investing in this field to enable for a more improved quality of life for cancer patients; this can be a main factor in saving lives worldwide.

## Author Contributions

**Aya Sedky Adly**: data curation, conceptualization, investigation, methodology, visualization, and writing original draft. **Guillaume Cartron**: revising, coordinating, and editing the manuscript. **Afnan Sedky Adly**: data curation, conceptualization, investigation, methodology, visualization, and writing original draft. **Jean–Christophe Egea**: revising, coordinating, and editing the manuscript. **Pierre–Yves Collart Dutilleul**: revising, coordinating, and editing the manuscript. **Mahmoud Sedky Adly**: data curation, conceptualization, investigation, methodology, visualization, and writing original draft. **Martin Villalba**: revising, coordinating, and editing the manuscript. All authors gave final approval.

## Ethics Statement

The authors have nothing to report.

## Conflicts of Interest

The authors declare no conflicts of interest.

## Data Availability

The authors have nothing to report.
